# Acid-sensing ion channels and downstream signalling in cancer cells: is there a mechanistic link?

**DOI:** 10.1007/s00424-023-02902-z

**Published:** 2024-01-04

**Authors:** Stefan Gründer, Jakob Vanek, Karolos-Philippos Pissas

**Affiliations:** Institute of Physiology, RWTH Aachen University, Pauwelsstraße 30, 52074 Aachen, Germany

**Keywords:** Ca^2+^ signalling, Carcinoma, Epithelial-mesenchymal transition, Necroptosis, pH sensor, RIPK1, ROS, Tumour microenvironment

## Abstract

It is increasingly appreciated that the acidic microenvironment of a tumour contributes to its evolution and clinical outcomes. However, our understanding of the mechanisms by which tumour cells detect acidosis and the signalling cascades that it induces is still limited. Acid-sensing ion channels (ASICs) are sensitive receptors for protons; therefore, they are also candidates for proton sensors in tumour cells. Although in non-transformed tissue, their expression is mainly restricted to neurons, an increasing number of studies have reported ectopic expression of ASICs not only in brain cancer but also in different carcinomas, such as breast and pancreatic cancer. However, because ASICs are best known as desensitizing ionotropic receptors that mediate rapid but transient signalling, how they trigger intracellular signalling cascades is not well understood. In this review, we introduce the acidic microenvironment of tumours and the functional properties of ASICs, point out some conceptual problems, summarize reported roles of ASICs in different cancers, and highlight open questions on the mechanisms of their action in cancer cells. Finally, we propose guidelines to keep ASIC research in cancer on solid ground.

## Acidosis as a characteristic of the tumour microenvironment

Cancer is a complex and polymorphic disease characterized by significant differences between cancer and non-cancer cells. While carcinogenesis has already been extensively studied on a cellular level over the years, recent attention has increasingly focused on the unique tumour microenvironment (TME) that differs substantially from its physiological counterpart. The TME plays a crucial role in cancer development, progression, and behaviour [65]. The most-extensively studied characteristic of the TME is hypoxia, defined as a decreased oxygen supply at tissue, resulting from inadequate vascularization of rapidly proliferating tumour cells [35]. Another common, yet less explored, characteristic is its acidity: Cancer cells are typically surrounded by a milieu containing a substantially higher concentration of protons. Tumour tissue has been reported to exhibit pH values as low as 6.6 or even lower [30, 76].

**Fig. 1:**
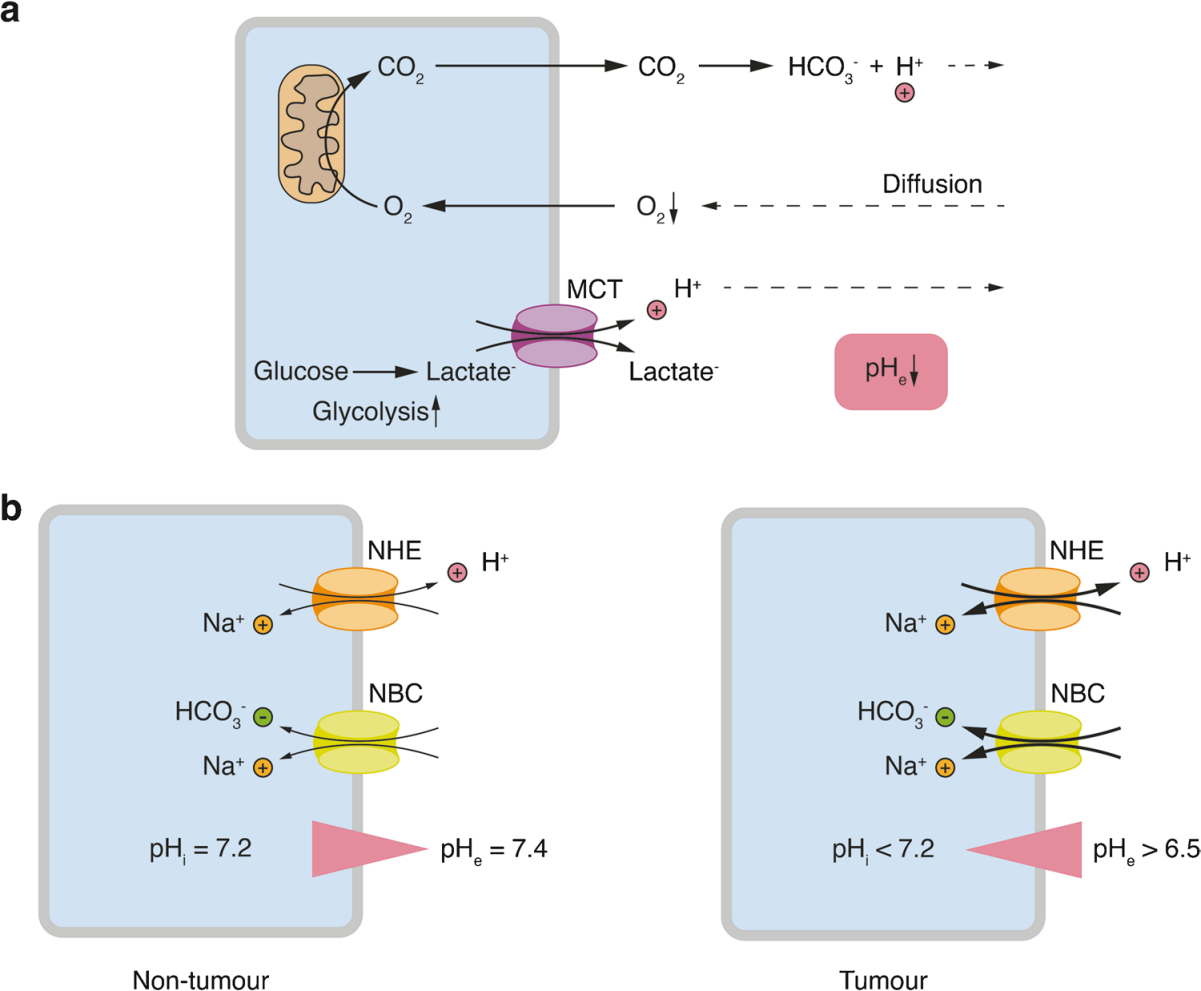
Disturbed acid–base regulation in cancers. **a** The increased metabolic activity of cancer cells leads to increased CO_2_ production and generation of protons. Due to increased O_2_ consumption and decreased delivery of O_2_, the TME often becomes hypoxic and glycolysis is increased; the Warburg effect further increases glycolysis and production of lactate and protons. Due to the increased diffusion distance, protons accumulate in the TME and pH_e_ decreases. MCT, monocarboxylate transporter. **b** Left, in non-tumour cells, the proton concentration within the cells is slightly higher than outside of the cells. Right, in cancer cells, the activity of acid-extruding transporters is increased and the proton gradient can be inversed. However, the pH_i_ is often still more acidic than in non-tumour cells. The actual pH values vary depending on the local position of a cell within a tumour. NHE, Na^+^-H^+^-exchanger; NBC, Na^+^-bicarbonate cotransporter

In general, pH homeostasis is crucial for cellular function, as alterations in pH can impact the function and activity of enzymes, ion channels, transporters, and other cellular proteins. Acidity is a characteristic feature of various pathophysiological states, not only cancer. Particularly in cancer, multiple mechanisms contribute to an increased intracellular proton concentration (decrease of the intracellular pH, pH_i_) as well as to the extrusion of protons. Given that pH regulation is a highly dynamic process, protons are constantly released to and removed from the extracellular space. At a certain point in carcinogenesis, proton release into the extracellular space surpasses its removal, primarily due to inadequate vascularization. This imbalance finally leads to an accumulation of protons in the extracellular compartment, causing a drop of the extracellular pH (pH_e_) [32] (Fig. 1a). Consequently, an acidic TME is a key characteristic in cancer, exhibiting a heterogeneous spatial pH distribution within the tumour mass [37], so that some cancer cells face an acidic pH_e_ while others do not.

There are several reasons for the increased intracellular release of protons, including enhanced glycolysis and mitochondrial respiration. But enhanced glycolysis appears to be characteristic for cancer cells: Their increased growth, especially within the core of the tumour mass, results in limited access to oxygen due to an inadequate vascularization, leading to hypoxia [75]. Hypoxia shifts cellular metabolism in both cancer and non-cancer cells towards glycolysis. This adaptation allows cells to account for their ATP requirements without relying on oxygen molecules. In contrast to 38 molecules of ATP per glucose produced through oxidative phosphorylation, the 2 molecules of ATP per glucose resulting from the Pasteur effect seem to be rather inefficient and contribute to the increased acid production to meet cellular ATP demands [45] (Fig. 1a). Remarkably, while most non-tumour cells predominantly shift their metabolism towards lactic acid fermentation during oxygen deprivation, cancer cells as well as highly proliferating non-cancer cells tend to exhibit this behaviour even without limited access to oxygen, referred to as Warburg effect [29].

Hence, it is interesting that cancer cells respond differently to an acidic TME than non-tumour cells and generally exhibit better resistance to this extracellular stressor. To understand this phenomenon, it is essential to appreciate that pH_i_ and pH_e_ are tightly coupled, with an electrochemical driving force favouring intracellular acidification relative to the extracellular compartment. For a pH_i_ of 7.2, which corresponds to 63 nM protons, and a pH_e_ of 7.4, which corresponds to 40 nM protons, the equilibrium potential E_H_ for protons is ~ -12 mV, indicating an inwardly directed driving force for protons at a membrane potential more negative than E_H_. To avoid a drastic drop of the pH_i_, which is a potent inductor of apoptosis, active acid extrusion mechanisms are indispensable. These mechanisms involve either extruding protons, for example facilitated by Na^+^/H^+^-exchangers (NHEs) or monocarboxylate transporters (MCTs), or importing HCO_3_^−^-ions, for example through Na^+^/HCO_3_^−^-cotransporters (NBCs), which then buffer the excess of intracellular protons. Consequently, compared to typical physiological conditions with a pH_e_ ~ 7.4 and a pH_i_ ~ 7.2, this gradient is often reversed in cancer cells [30] (Fig. 1b). In cancer cells, acid-extruding mechanisms are typically upregulated compared to their non-tumour counterparts [70], contributing to the reversed pH gradient. However, it is crucial to point out that within the TME this is a rather relative phenomenon, resulting in cancer cells still exhibiting a lower or at least equal pH_i_ compared to non-cancer cells in their typical microenvironment (Fig. 1b).

Although still not as extensively studied as the hypoxic TME, there is a growing number of studies focusing on the impact of an acidic TME on cancer cell behaviour [72]. Recent publications demonstrate that alterations of the pH_e_ affect different aspects of cancer cell behaviour, including migration [19], invasion [26], metastatic potential [40], proliferation, stemness and cell death [18], and can also promote somatic evolution of cancer cells [64]. However, the precise mechanisms by which cancer cells sense pH alterations remain poorly understood. There are various possibilities on how cells can sense the pH_e_. One possibility involves the coupling of the pH_i_ to the pH_e_: Secondary changes in pH_i_ could potentially impact entire metabolic pathways due to pH-optima of key enzymes, as shown for glycolysis [60]. Alternatively, intracellularly located direct proton or HCO_3_^−^-sensors, including the FAK-related kinase PYK2 [53] and the soluble adenylate cyclase [16] might act as sensors.

Given that the pH_e_ to pH_i_ gradient is often reversed in cancer cells [48], direct sensing of the pH_e_ may be more significant in cancer cells. Extracellular proton sensing has already been demonstrated for G-protein-coupled receptors, including GPR68, GPR4 and GPR5 [41], whereas the receptor protein tyrosine phosphatase-γ (RPTPγ) has recently been identified as a sensor for the extracellular HCO_3_^−^-concentration [99]. Another group of sensors, although less extensively studied, includes acid-sensing ion channels (ASICs). Their activation by extracellular protons and modulation by intracellular protons [52], make them interesting pH-sensing targets, particularly in cancer cells.

## Functional properties of acid-sensing ion channels

**Fig. 2:**
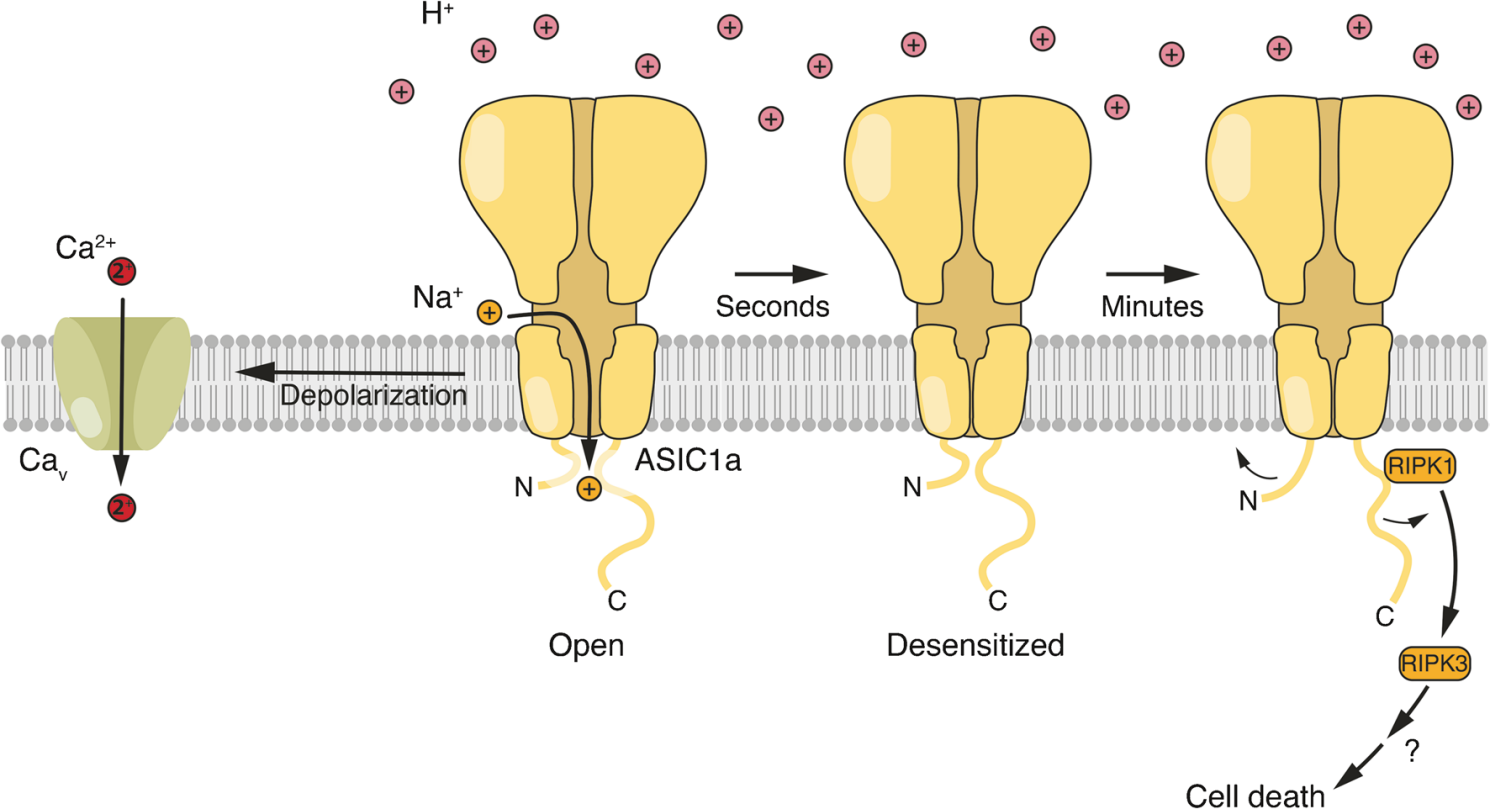
Ionotropic and metabotropic signalling of ASIC1a. A sudden drop in pH activates ASIC1a, leading to a transient influx of Na^+^ and depolarization of the cell. This depolarization could contribute to the excitatory postsynaptic potential at nerve cell-tumour cell synapses. In addition, it can trigger the activation of Ca_v_s and transient influx of Ca^2+^. Within seconds, ASIC1a desensitizes, and the pore is shut. This will also lead to the closure of Ca_v_s. It has been proposed that during sustained acidosis, the N and C termini of ASIC1a move apart, enabling the interaction of the C terminus with RIPK1 to trigger cell death

An ASIC subunit has two transmembrane domains and a large extracellular domain (ECD), to which protons and modulators bind. The relatively short N and C termini are in the cytoplasm (Fig. 2). Three ASIC subunits assemble into functional homo- or heterotrimers. ASICs are weakly selective Na^+^ channels (P_Na_/P_K_ = 5–14) [33] such that the opening of an ASIC depolarizes a cell.

There are six principal ASIC isoforms in humans, namely ASIC1a, ASIC1b, ASIC2a, ASIC2b, ASIC3, and ASIC4. Most studies to date have reported a role for ASIC1a in cancer cells, and a few for ASIC2a and ASIC3 as well. Homomeric ASIC1a and ASIC3 have high proton sensitivity: They start to be activated at pH 6.8—7.1 and are half-maximally activated at pH_50_ ~ 6.6 [33]. Homomeric ASIC2a has a much lower pH sensitivity (pH_50_ < 5.0) [4, 13, 42], but forms heteromers with ASIC1a and ASIC3. Heteromeric ASIC1a/2a and ASIC3/2a have a relatively low pH_50_ (pH_50_ < 6.0), but start to activate at pH ~ 6.7 [42, 93]. Thus, homomeric ASIC1a and ASIC3 and perhaps heteromeric ASIC1a/2a and ASIC3/2a are sensitive enough to sense the pH values that are typical for the acidic TME (pH 7.0 – 6.6; Table 1).

**Table 1:** pH-sensitivity of selected ASICs

	pH at which activation starts	pH of half-maximal activation
ASIC1a	6.8	6.5
ASIC3	7.1	6.6
ASIC1a/2a	6.7	5.8
ASIC3/2a	6.9	5.6

Note that reported pH-sensitivities vary with the expression system that was used. Moreover, not all values have been determined with human isoforms. Values for human homomeric ASIC1a are from reference [74], for human homomeric ASIC3 from [22], for rat heteromeric ASIC1a/2a from [42], and for rodent heteromeric ASIC3/2a from [93] and [36], respectively

In non-transformed tissues, ASIC expression is mainly confined to neurons. Homomeric ASIC1a and heteromeric ASIC1a/2a are the most abundant ASICs in the central nervous system [2, 5, 90], but ASIC1a/2a heteromers are probably less abundant in the human than in the rodent brain [22, 51], for which we have most data. ASIC3 is primarily expressed in the peripheral nervous system [39]. In the brain, ASIC1a localizes to the postsynaptic membrane [85, 95], and upon binding of protons, which are released during synaptic transmission, it opens and allows the transient influx of Na^+^, contributing to excitatory postsynaptic currents in different brain regions [23, 31, 46]. Thus, the main function of ASICs appears to be the mediation of phasic responses to synaptic fluctuations in the proton concentration. Intriguingly, in recent years it has become apparent that many tumours are innervated and that neuronal input drives tumour progression [56, 63, 77–79, 89]. Thus, it is an exciting possibility that ectopic expression of ASIC1a in tumour tissues contributes to the response of tumour cells to synaptically released protons. However, this hypothesis has not yet been explored.

ASIC1a may also localize to extra-synaptic sites, for example on the soma of neurons, where it could mediate tonic responses to the ambient proton concentration. The problem with this idea is that within a few seconds of binding of protons, ASIC1a enters a non-conducting, desensitized state, from which it can no longer be activated (Fig. 2). Even at a slightly reduced pH, ASIC1a enters the desensitized state without apparent opening, such that all channels are desensitized at pH < 7.0 [4]. This process, known as steady-state desensitization (SSD), severely limits the ability of homomeric ASIC1a to signal during sustained acidosis [33]. Recovery from desensitization (return to a closed conformation) requires that the pH returns to values > 7.0 [4]. Thus, ASIC1a is ideally suited for detecting transient acidification but not the acidic pH of the TME. The same applies for ASIC1a/2a. Nevertheless, so far, most studies on ASICs in cancer cells have explored the response of ASICs to sustained acidification. Only for homomeric ASIC3 and heteromeric ASIC3/2a, activation and SSD curves slightly overlap, generating a small sustained “window” current near pH 7.0 for ASIC3, and at slightly more acidic pH for ASIC3/2a [93]. This window current leads to a small but sustained influx of Na^+^, slightly depolarizing the cell. This is important, for example, for triggering cardiac pain associated with myocardial ischemia [71, 93].

Pharmacological inhibition is one way to test the involvement of ASICs in specific cellular events. The canonical ASIC inhibitor is amiloride. It provides a convenient, low-cost drug to test the involvement of ASICs. However, because amiloride is not specific for ASICs and has relatively low potency for ASICs (IC_50_ ~ 10 μM), it can only provide first and inconclusive evidence for the involvement of ASICs. In particular, it needs to be considered that amiloride also inhibits NHEs with a similar IC_50_ as ASICs [28]. Therefore, other, more specific ASIC inhibitors should also be used. The standard toxin that inhibits homomeric ASIC1a with high potency (IC_50_ ~ 5 nM) and relatively high specificity is psalmotoxin 1 (PcTx1) [14, 25]. In addition, the snail toxin MitTx is an ASIC1a agonist that can be used to activate ASIC1a homomers at neutral pH [8].

ASICs belong to the DEG/ENaC gene family [44]. Other members of this family in humans are the bile-acid-sensitive ion channel BASIC (also named INaC or ASIC5) [68, 88] and the α-, β-, γ-, and δ-subunits of the epithelial Na^+^ channel (ENaC). BASIC is relatively closely related to ASICs [86], but is insensitive to protons [87]. Although ASIC-BASIC heteromers have never been described, they cannot be formally excluded. In contrast, recent phylogenetic analyses have shown that ENaC belongs to a clade of the DEG/ENaC gene family that is separated by > 500 million years of evolution from the clade containing ASICs [1, 24]. Because both subgroups have evolved independently for such a long time, it is unlikely that they still form functional heteromers. Although such ASIC-ENaC heteromers have been postulated in some cancer cells (see below), their existence is questionable in the absence of robust experimental support.

## How could the activation of ASICs trigger downstream signalling in cancer cells?

The main mechanistic link between ASIC activation and proliferation, migration, and invasion of cancer cells, which has been postulated so far, is Ca^2+^ signalling (see below). Homomeric ASIC1a and human homomeric ASIC1b are the only ASICs that are also permeable to Ca^2+^ [6, 39, 81]. The Ca^2+^ permeability of ASIC1a homomers is low (P_Na_/P_Ca_ ~ 20) [6], however, and the effect of ASIC1a activation on the intracellular Ca^2+^ concentration ([Ca^2+^]_i_) is often overestimated. Even though the Ca^2+^ permeability of human ASIC1b homomers seems to be higher (P_Na_/P_Ca_ ~ 2.5) [39], the significance of this finding remains unclear, as ASIC1b has not yet been described in cancer cells. Most studies report that the activation of ASIC1a homomers is insufficient to substantially increase [Ca^2+^]_i_ by itself [67, 73, 96], but may do so via the activation of voltage-gated Ca^2+^ channels (Ca_v_s) [58, 96] (Fig. 2). Therefore, while opening homomeric ASIC1a might increase the local Ca^2+^ concentration in a nanodomain close to the channel, the bulk of Ca^2+^ influx occurs via Ca_v_s and relies on the presence of these channels in cancer cells. Since carcinomas are derived from epithelial cells, which are typically non-excitable, it is important to test for the presence of Ca_v_s in carcinoma cells when an increase in [Ca^2+^]_i_ is claimed. In any case, due to the desensitization of ASIC1a and inactivation of Ca_v_s, the influx of Ca^2+^ triggered by ASIC1a will be transient. Moreover, it is possible that Ca_v_s, even if present, are inactive at the depolarized membrane potential that is typical for cancer cells [94].

It is also important to keep in mind that Ca^2+^ signals in cells are typically not only transient but also local. Ca^2+^ diffusion within a cell is strongly limited by Ca^2+^-binding proteins. In addition, there are efficient ways to remove Ca^2+^ from the cytoplasm, such as the plasma membrane Ca^2+^-ATPase and the Na^+^-Ca^2+^-exchanger (NCX). Although it has been reported that ASIC1a activation induces Ca^2+^ waves that propagate to mitochondria [3], our understanding of the temporal and spatial Ca^2+^ signals triggered by ASIC opening in cells is limited and it is unclear how the transient influx of Ca^2+^ triggered by ASIC activation can lead to dysregulation of Ca^2+^ signalling, which needs to be assumed to explain at least some of the presumed effects of ASICs on cancer cells.

Recently, a completely new metabotropic function, which is independent of ion conduction, was proposed for ASIC1a [84]. Several independent studies have reported that sustained activation of ASIC1a, for example during the acidosis associated with ischemic stroke, leads to neurodegeneration [11, 92]. But how can a completely desensitizing ion channel signal sustained acidosis and induce cell death? Interestingly, it has recently been reported that upon prolonged activation, ASIC1a directly interacts and activates receptor interacting protein kinase 1 (RIPK1) [84]. RIPK1 is known to be part of the necroptosis pathway [17, 50], and it has been proposed that activation of RIPK1 by ASIC1a leads to neurodegeneration. Moreover, it has been proposed that the ASIC1a-RIPK1 interaction is triggered by slow conformational changes in the cytoplasmic termini of ASICs during sustained acidosis that break electrostatic interactions between the cytoplasmic N and C termini, liberating the C terminus for interaction with RIPK1 [83] (Fig. 2). Interestingly, pretreatment with inhibitors of RIPK1 was necessary to prevent acid-induced cell death, suggesting activation of RIPK1 at the onset of acidotoxicity in neurons [84].

However, experimental support for this model is ambiguous. One study using fluorescence resonance energy transfer (FRET) to interrogate motions of the cytoplasmic N and C termini of ASIC1 did not find evidence for the predicted lateral movement (Fig. 2), but found that they slightly moved toward the plasma membrane upon extracellular acidification [20]. Another study found that the N and C termini are not close enough to each other to directly interact [21]. Although these methods have limitations, they highlight that we still need to better understand the dynamics of the ASIC1a-RIPK1 interaction. Moreover, it is unclear whether activation of RIPK1 by ASIC1a triggers necroptosis or another cell death pathway (see also below) [18]. Thus, many aspects of the sustained, metabotropic signalling by ASIC1a remain unclear.

**Fig. 3:**
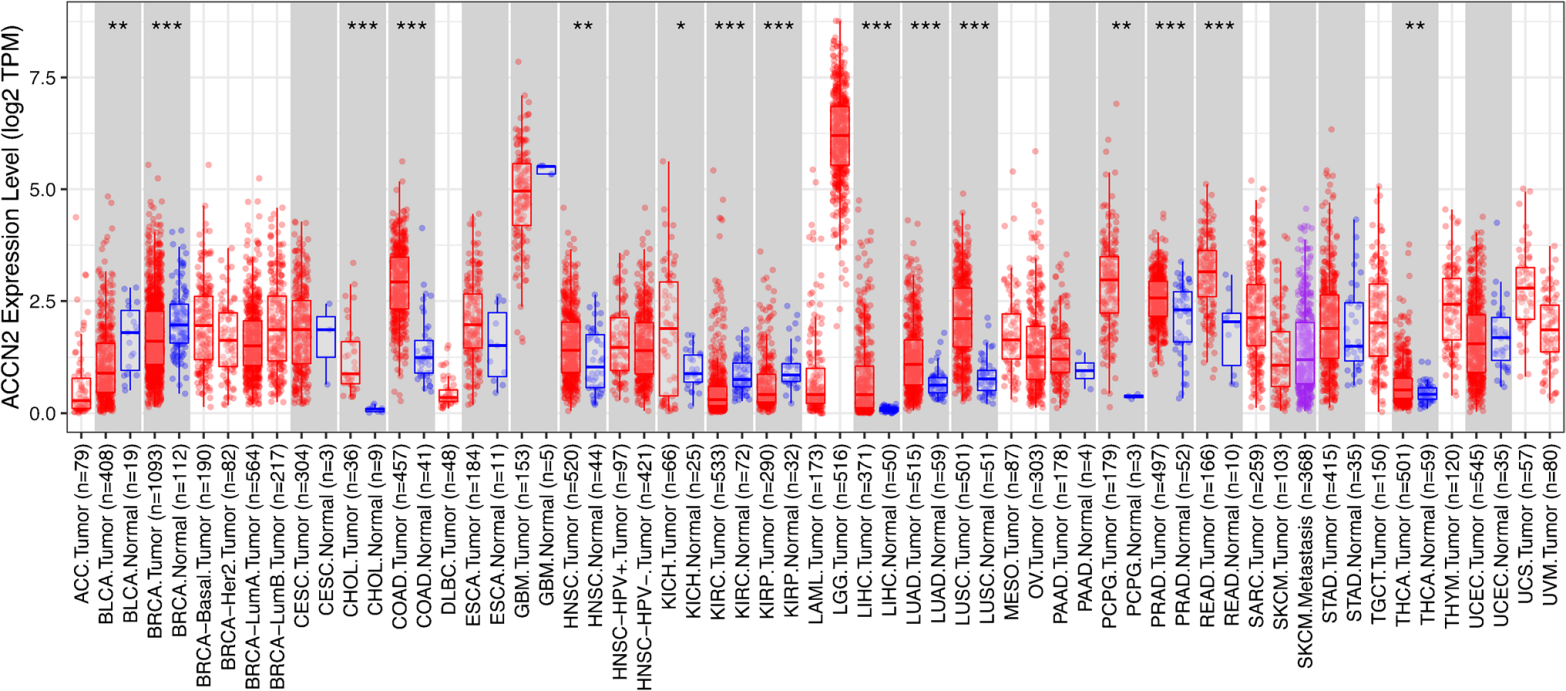
*ASIC1* (alias: *ACCN2*) expression level in different cancer types. Differential expression of *ACCN2* (*ASIC1*) between tumor and adjacent normal tissues was analyzed using data from “The Cancer Genome Atlas” (TCGA), accessed through TIMER2.0 [54] on November 15, 2023. *ACCN2* is expressed across multiple cancer types, with the highest expression in Lower Grade Glioma (LGG) and Glioblastoma (GBM). Additionally, *ACCN2* shows a significantly elevated expression in breast invasive carcinoma (BRCA), cholangiocarcinoma (CHOL), colon adenocarcinoma (COAD), head and neck squamous cell carcinoma (HNSC), kidney chromophobe carcinoma (KICH), liver hepatocellular carcinoma (LIHC), lung adenocarcinoma (LUAD), lung squamous cell carcinoma (LUSC), pheochromocytoma and paraganglioma (PCPG), prostate adenocarcinoma (PRAD), rectum adenocarcinoma (READ) and thyroid carcinoma (THCA). Conversely a statistically significant decrease in *ACCN2* expression was observed in bladder urothelial carcinoma (BLCA), kidney renal clear cell carcinoma (KIRC) and kidney renal papillary cell carcinoma (KIRP) compared to non-tumor tissue. * *p* < 0.05, ** *p* < 0.005, *** *p* < 0.001)

In summary, while ASIC1a may have metabotropic functions in addition to its ionotropic functions, to date, only a direct interaction with RIPK1 to induce cell death has been reported. Moreover, how ASIC1a could selectively initiate specific cellular signalling pathways by triggering Ca^2+^ influx is largely unknown. With these ideas in mind, we will now review the reported roles of ASICs in different types of cancer. We will largely exclude reports from our review in which the role of ASICs has been investigated only at neutral pH 7.4, because the downstream signalling involved is obscure. Figure 3 provides an overview of the mRNA abundance of ASIC1a in different cancer types (red) and normal tissue (blue).

## ASICs in brain cancer – increased migration vs. increased cell death

Already > 20 years ago, the presence of a small, constitutive, amiloride-sensitive cation current was reported in different glioblastoma multiforme (GBM) cell lines as well as in primary GBM cultures and in freshly isolated medulloblastoma (MB) cells. Moreover, while ASIC1a was highly expressed in these cells, ASIC2 was often downregulated [7, 10]. However, the evidence that this constitutive current was related to ASICs was inconclusive, and rested mainly on its sensitivity to amiloride (IC_50_ 10–30 μM) [7, 10] and to PcTx1 [9]. On the other hand, the findings that the K^+^ fraction of this current was approximately fourfold higher than the Na^+^ fraction [9] and that this current was present at pH 7.4 speak against a contribution of ASICs. Nevertheless, in subsequent studies, it was reported that the inhibition or knockdown of ASIC1a or epithelial sodium channel (ENaC) subunits impaired the proliferation and migration of GBM cells in vitro [43, 66]. The authors proposed that the cells express an ASIC1a-ENaC heteromer [7, 43, 66] and that its aberrant and constitutive activity is caused by a lack of ASIC2 surface expression [80]. Surprisingly, however, this channel had not been reconstituted in heterologous expressions systems and these studies never attempted to assess the presence of a typical ASIC current by application of low pH. Therefore, the evidence for the existence of ASIC-ENaC heteromers remains inconclusive and, in the absence of robust experimental support, highly speculative. In addition, the effects of this small cation conductance on migration and proliferation were all measured at pH 7.4, implying that ASIC-ENaC heteromers would not serve as proton sensors in GBM and other brain tumour cells.

Glioma cell lines cultured in serum-containing medium, which are often used in in vitro studies such as these early studies on ASICs in GBM, are poorly representative of primary tumours [49]. In contrast, glioblastoma stem cell lines (GSCs), cultured in serum-free conditions as tumourspheres, better represent the primary GBM [47]. Functional analysis of ASICs in such GSCs revealed the presence of typical ASIC currents, mediated by ASIC1 and ASIC3; like previously reported for GBM cells, ASIC2 was downregulated in GSCs [73]. In contrast, a constitutive amiloride-sensitive Na^+^ current could not be detected [73]. While slight acidosis (pH 6.6) strongly increased migration of GSCs in a tumoursphere outgrowth assay in vitro, neither pharmacological inhibition or genetic knockout of ASIC1a nor overexpression of ASIC2a affected migration of GSCs in vitro [19]. The increased migration in acidic medium was rather mediated by phosphoinositide 3-kinase (PI3K) [19], a known mediator of cancer cell migration. Another study using GBM cells cultured in serum-containing medium, also reported functional expression of a typical ASIC current, but found that inhibition or knockdown of ASIC1a reduced the increase in migration at slightly acidic pH (7.0) [69]. Thus, there is controversial evidence on the role of ASICs in migration of GBM cells in vitro. However, recent studies did not confirm a role of ASIC1a in proliferation of GBM cells or GSCs [18, 69].

Which other role could ASICs play in GBM? Because sustained activation of ASIC1a induces neurodegeneration, it has been investigated if it does so in GSCs as well. Surprisingly, it was indeed found that ASIC1a activation at slight acidosis (pH 6.6) significantly reduced tumoursphere formation and induced necrotic morphology of the tumour cells. In contrast, sphere size or proliferation was not affected [18], suggesting that acidosis induces cell death at the onset of acidification, in which GSCs appear to be vulnerable to cell death induction. Whether this mechanism is relevant for the evolution of a tumour in situ is unknown, and future studies need to address this question in xenograft animal models. Furthermore, it is unclear whether this mode of cell death will be beneficial or detrimental for patients, given the ambivalent nature of necroptosis and its possible pro- and anti-tumoural effects [62].

Interestingly, a recent study, which has not yet been peer-reviewed, reported reduced tumour growth and prolonged survival of mice after transplanting the murine glioma cell line GL261 into the brain of mice with ASIC1a gene deletion [59]. Importantly, ASIC had been knocked-out in the host tissue and not in the tumour cells. ASIC expression in GL261 cells was not assessed. The authors proposed that the acidic TME increased ASIC signalling also in neighbouring neurons leading to an increased connectivity between neurons and glioma [59], which is known to drive glioma progression [77–79]. However, the mechanisms and signalling pathways underlying this regulatory role of ASIC1a on neuron-glioma connectivity remained unclear.

The expression of functional ASIC1a homomers has recently also been reported for DAOY cells, which are derived from the paediatric tumour MB; ASIC2 and ASIC3 were not expressed [61]. Furthermore, it was found that DAOY cells were resistant to acid-induced cell death and that they expressed low levels of RIPK3, a kinase downstream of RIPK1 in the necroptosis pathway. Strikingly, overexpressing RIPK3 in DAOY cells rendered them vulnerable to acid-induced cell death in an ASIC1a-dependent fashion [61]. Thus, it appears that sustained activation of ASIC1a can, in principle, induce regulated cell death also in MB cells.

In summary, although the expression of ASICs in malignant gliomas has been increasingly studied over the recent years, their exact functions remain unclear. While early studies suggested a role for unconventional ASIC-ENaC heteromers in pro-tumoral proliferation and migration, more recent studies could not confirm these observations. Possible anti-tumoral effects through an ASIC1a-mediated necroptosis-related cell death pathway have instead been proposed. While ASICs are also expressed in MB tissue, their expression in other types of brain tumours, such as meningiomas or ependymomas has not yet been investigated.

## ASICs in breast and prostate cancer – increase of reactive oxygen species

**Fig. 4:**
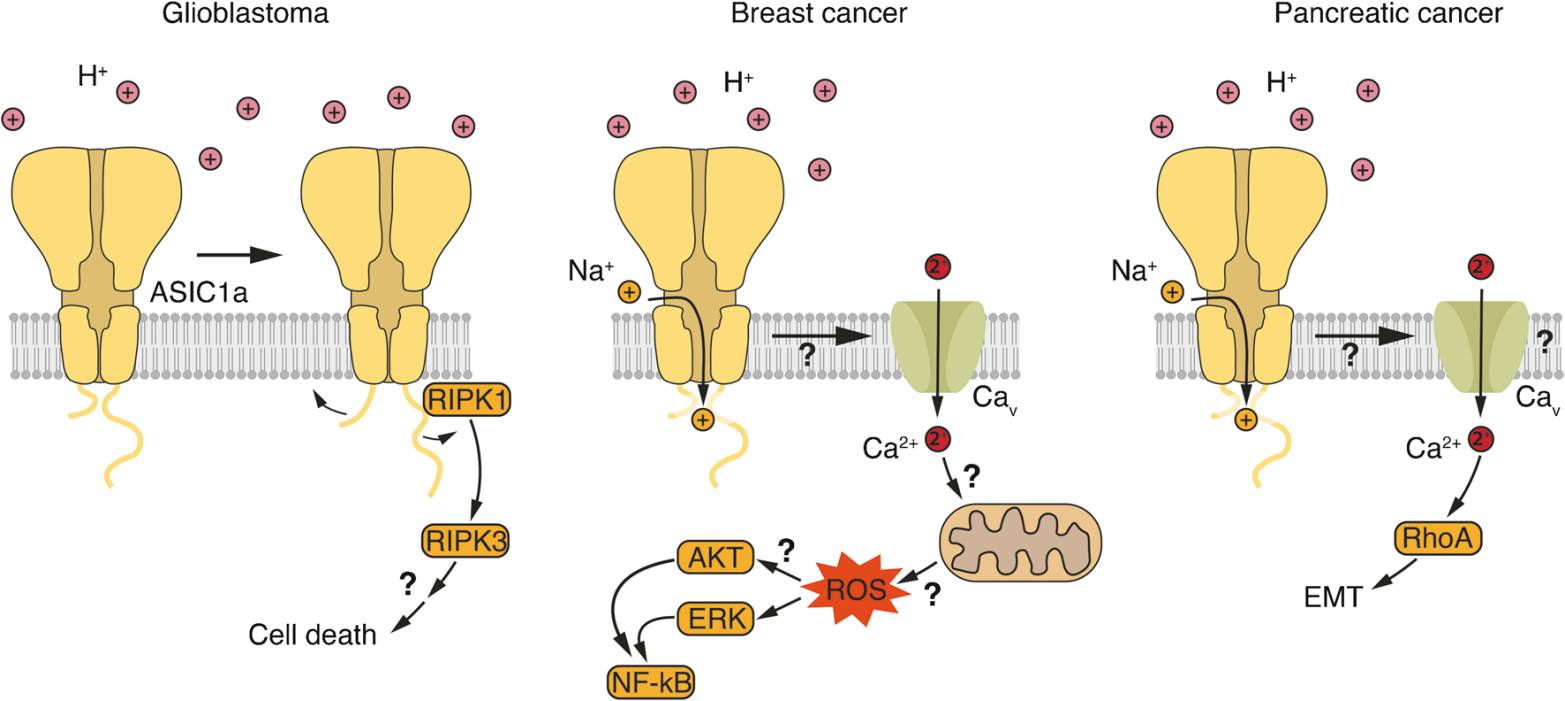
Proposed signalling pathways of ASIC1a in different cancers. In glioblastoma stem cells, it appears that under certain conditions ASIC1a can induce a programmed cell death involving RIPK1. For breast cancer cells, it has been reported that ASIC1a triggers the formation of ROS and ensuing activation of AKT and NF-κB. A similar pathway may be active in prostate cancer cells. For pancreatic cancer, it has been proposed that ASIC1a triggers the activation of RhoA and the ensuing EMT. For details, see text

One of the first studies to examine the role of ASICs in breast cancer found that ASIC1a is overexpressed in this epithelia-derived carcinoma [34]. It was shown that acidosis (pH 6.6) increases the levels of reactive oxygen species (ROS) in MCF-7 and LM-4142 breast cancer cell lines in an ASIC1a-dependent manner. Moreover, it was shown that LM-4142 cells express functional ASIC (amplitude: > 1 nA at pH 5), that acidosis increases [Ca^2+^]_i_ in an ASIC1-dependent manner, and that Ca^2+^ chelation prevents the formation of ROS in these cells. It has been proposed that acidosis activates ASIC1a, leading to an influx of Ca^2+^, probably via Ca_v_s. The increased [Ca^2+^]_i_ would trigger ROS formation, which would then activate the protein kinases AKT and ERK1/2 and finally nuclear factor κB (NF- κB) [34] (Fig. 4). These effects increased the invasion of LM-4142 cells in an in vitro Matrigel assay and increased tumour growth and metastasis in a xenograft mouse model [34]. Although not all causal relationships had been delineated, this study proposed a clear mechanism for the role of ASIC1a in breast cancer cell lines. The critical link between ASIC activation and downstream signalling is Ca^2+^ signalling and ROS production. While the relationship between dysregulated Ca^2+^ signalling and increased ROS production is well established [38, 55], the main source of ROS are the mitochondria [27, 82], and it is surprising that the transient and local increase in [Ca^2+^]_i_ in the cytosol, which is expected from ASIC1a activation (the time course of the Ca^2+^ signal was not reported in this study), was sufficient for robust ROS formation in breast cancer cells. While a recent study reported that ASIC1a activity increases [Ca^2+^] in mitochondria of mouse neurons, it also reported that ASIC1a activation decreased rather than increased ROS production [3]. Therefore, ROS formation as a consequence of ASIC1a activation and its mechanistic details need to be further explored.

In a follow-up study, it was shown that 22Rv1 prostate cancer cells express ASIC1a, and that while AKT is constitutively active in these cells, acidosis (pH 6.6) generates ROS and activates ERK1 in an ASIC1a-dependent manner [12]. ERK would then phosphorylate IκB kinase (IKK), leading to the degradation of IκB and activation of NF-κB. These effects increased invasion in an in vitro Transwell assay [12]. Again, the critical link between ASIC1a activation and ROS production requires further confirmation.

## ASICs in pancreatic cancer – activation of RhoA

It was reported that PANC-1 and BxPC-3 pancreatic cancer cells express functional ASIC1a and ASIC3 [101]. The ASIC current had a small amplitude (< 100 pA at pH 6.4), however, was only 50% blocked by 100 μM amiloride, and did not have the typical transient kinetics of ASIC1a and ASIC3 currents [101]. Overall, there is only weak evidence for the expression of functional ASICs in these cell lines. Nevertheless, downregulation of ASIC1 or ASIC3 expression by siRNA in these cells completely rescued the increased invasion at acidic pH (pH 6.4) in an in vitro Transwell assay and the increased migration in an in vitro scratch assay. Similarly, amiloride completely rescued the increased migration of the cells [101]. It is surprising that while knockdown of either ASIC1a or ASIC3 should only partially reduce ASICs expression and while amiloride only partially reduced the small ASIC currents, each manipulation separately led to a complete rescue of increased invasion and migration. Moreover, it was reported that the expression of mesenchymal markers increased at pH 6.4 and that this increased expression was completely abolished by knockdown of either ASIC1 or ASIC3 by siRNA [101]. Acidic pH induced a Ca^2+^ signal that lasted for 60 – 350 s, depending on the pH and the cell line. However, this Ca^2+^ signal was only modestly reduced by knockdown of either ASIC1 or ASIC3 or by amiloride [101]. Moreover, it was found that acidosis increases the activity of the small G protein RhoA, which is involved in cytoskeletal regulation. Although this increased RhoA activity was abolished by knockdown of either ASIC1 or ASIC3, by amiloride, or by chelating Ca^2+^ [101], the mechanistic details linking ASIC activation, Ca^2+^ signalling, and RhoA activation remain unclear (Fig. 4). Knockdown of RhoA reduced the increased invasion and migration, and reversed the increased expression of mesenchymal markers [101]. In summary, although this study provided a first indication that ASIC1a and ASIC3 might be involved in epithelial-mesenchymal transition (EMT) in pancreatic cancer cells, many questions regarding the specific role of ASICs remain open.

## ASICs in colorectal cancer – activation of NFAT

It has been reported that ASIC2a is highly expressed in some colorectal cancer cell lines, but weakly expressed in others [100]. Overexpression of ASIC2a in weakly expressing cells increased invasion in a Transwell assay at pH 6.5 (but not at pH 7.4), whereas downregulating it in highly expressing cells decreased invasion at pH 6.5. Moreover, overexpression of ASIC2a increased and downregulation decreased proliferation and colony formation, respectively, surprisingly also at neutral pH 7.4 [100]. Moreover, it was shown that nuclear translocation of nuclear factor of activated T cells (NFAT1) increased after overexpression of ASIC2a and decreased after its downregulation, and it was proposed that an increase in [Ca^2+^]_i_ by activation of ASIC2a triggered nuclear translocation of NFAT1 [100]. ASIC currents were not recorded in this study, and it is unclear why the expression of the ASIC2a subunit, which has low H^+^-sensitivity, should increase [Ca^2+^]_i_. In addition, Ca^2+^ signals were not measured. Therefore, the mechanistic link between ASIC2a expression and NFAT activation remains to be elucidated.

## ASICs in lung cancer – increased proliferation and migration

It was reported that ASIC1, ASIC2, and ASIC3 are expressed in A549 lung cancer cells [91]. Typical transient ASIC currents were observed in these cells (~ 1 nA at pH 6.0) and were partially sensitive to PcTx1. Surprisingly, acidosis (pH < 7.0) not only increased migration of these cells in a scratch assay but also metabolic activity as measured via an MTT assay. Moreover, overexpression of ASIC1a increased whereas PcTx1 reduced proliferation and migration at pH 6.0 [91]. Finally, pH 6.0 induced a Ca^2+^ signal in these cells, which was reduced by PcTx1 [91] and it was proposed that ASIC activation induced the Ca^2+^ signal that then led to increased proliferation and migration.

## ASICs in hepatocellular carcinoma – activation of PI3K and AKT

Two independent studies reported that ASIC1a is expressed in hepatocellular carcinoma (HCC) cell lines and that it is upregulated in drug resistant cells; inhibition of ASICs by amiloride or knockdown of ASIC1a by shRNA enhanced chemosensitivity whereas overexpression of ASIC1a enhanced chemoresistance of HCC cells [97, 98]. Moreover, it was reported that pH 6.5 induced a Ca^2+^ signal in HCC cells, which was inhibited by amiloride but not the Ca_v_-inhibitor verapamil [98]. It was shown that ASIC1a knockdown reduced p-AKT in HCC cells and it was proposed that direct Ca^2+^ influx via ASIC1a activated PI3K [98]. It remained unclear, however, how knockdown of ASIC1a in cells kept at neutral pH can reduce phosphorylation of AKT [98]. Moreover, it was reported that ASIC1a promoted EMT in HCC cells, and it was proposed that EMT was mediated via the AKT/GSK3β/Snail pathway [97]. Increasing the complexity of the proposed effects, a different study reported an inverse cascade where in hepatic stellate cells, the PI3K/AKT pathway increases ASIC1a surface expression enhancing endoplasmic reticulum stress [102]. In another study, it was found that increased migration of liver cancer cells in an in vitro scratch assay and increased invasion in a transwell assay were reduced by PcTx1 or by knockdown of ASIC1a via shRNA. Overexpression of ASIC1a had the inverse effect [97]. It was proposed that ASIC1a activated the PI3K/AKT/MTOR pathway to induce the secretion of matrix metalloproteinases (MMPs) [97]. In this study, no ASIC currents or Ca^2+^ signals elicited by low pH were recorded and the mechanistic details of how ASIC activation might trigger the downstream effects were not elucidated.

## ASICs in gastric carcinoma

Using the gastric carcinoma cell line AGS, it was found that ASIC1a knock-down by shRNA reduced migration in a scratch assay at pH 7.4 and pH 6.5 and reduced tumour weight in a xenograft mouse model [15]. No mechanism for the observed effects was reported.

## Summary

In several cell models of diverse types of cancer, a role for ASIC1a in proliferation, migration and invasion has been proposed. Overall, a plethora of signalling cascades have been reported to be activated downstream of ASIC activation in different carcinomas – increase in ROS, activation of RhoA, NFAT, PI3K, or AKT—all due to an increase in [Ca^2+^]_i_ triggered by ASICs. The diversity of these effects all induced by supposedly transient Ca^2+^ signals shed some doubt on their specificity. Moreover, in vitro results have been obtained using established cell lines that grow as monolayers in serum-containing medium. These cells do not well represent the tumour heterogeneity and biology. Therefore, we need to test the role of ASICs also in more advanced models, such as tumourspheres and, if feasible, organoids. As an exception, recent studies using GSC tumourspheres did not find any role for ASICs in proliferation and migration [18, 19], but rather an induction of a regulated cell death pathway [18].

## Guidelines to address the challenges and conceptual problems of ASIC research in cancer

To strengthen the mechanistic link between ASIC activation and downstream signalling in cancer, we propose the following guidelines for studies addressing the mechanistic role of ASICs in cancer:

1. When the expression of an *ASIC* gene is assessed by qPCR, it should be normalized to an appropriate housekeeping gene and these values should be reported to allow the assessment of the absolute expression level. It is insufficient to only report *ASIC* expression at different pH normalized to expression at pH 7.4.
2. Western blots and immunocytochemistry are, in principle, appropriate methods to test for the presence of an ASIC, but it should be kept in mind that antibodies are often unspecific and, therefore, whenever possible, good controls (for example knock-out cells) should be employed.
3. Functional ASICs should be electrophysiologically characterized in the target tissue or cell.
4. The ASIC subtypes involved in functional ASICs should be identified by their electrophysiological characteristics and by pharmacological tools.
5. To proof the involvement of ASICs in a specific cellular event (for example, proliferation or migration), more than one inhibitor should be used. In addition, ideally, the outcome of disruption of the respective ASIC gene, for example using CRISPR-Cas9, should be assessed. Successful knockout or knockdown (via shRNA) is ideally verified by electrophysiology.
6. The functional role of ASICs for a cancer/cell should be addressed at acidic pH, in the range between 7.3 and 6.5 and the pH of the cell culture should be controlled following evidence-based guidelines [57].
7. If increases of [Ca^2+^]_i_ are proposed to be involved in the function of an ASIC, they should be directly demonstrated using Ca^2+^ imaging and the triggering function of ASIC1a (or other ASICs) for Ca^2+^ signals should be demonstrated with appropriate inhibitors of ASIC1a.
8. The presence of active Ca_v_s in cancer cells should be tested by application of a high K^+^-solution to depolarize the cell and the contribution of Ca_v_s to Ca^2+^ signals should be tested with appropriate inhibitors.
9. Cell models should be used which are as representative of the parental tumour as possible. In addition, the expression of a respective ASIC subunit in a certain tumour type should be ascertained by analysis of online databases containing expression data from tumour tissue.
10. If Kaplan–Meier analysis is performed, it should be clearly indicated what “High expression” and “Low expression” means. Ideally, the two groups are separated at the median expression level.

## Conclusions

In summary, while an increasing number of studies report a role for ASICs in proliferation, migration, death, and even drug resistance of cancer cells, the signalling pathways that have been proposed to be involved in these effects are surprisingly diverse and heterogenous and the evidence linking ASIC activation to downstream signalling events is often surprising and not sufficiently robust. Therefore, our understanding of ASIC for downstream signalling cascades needs to be increased and the mechanistic role of ASICs in tumour cell function needs to be further established before we can evaluate the significance of ASICs as a target in cancer cells.

## Data Availability

No datasets were generated or analysed during the current study.
